# Crosstalk between Inflammation and Hemorrhage/Coagulation Disorders in Primary Blast Lung Injury

**DOI:** 10.3390/biom13020351

**Published:** 2023-02-10

**Authors:** Junfeng Li, Jianfeng Zhang, Mingyu Shi, Sifan Yu, Mengjun Ji, Yangfan Liang, Xiangyan Meng

**Affiliations:** 1 Institute of Disaster and Emergency Medicine, Tianjin University, Tianjin 300072, China; 2 Tianjin Key Laboratory of Disaster Medicine Technology, Tianjin 300072, China; 3 Wenzhou Safety (Emergency) Institute, Tianjin University, Wenzhou 325000, China; 4 Key Laboratory of Medical Rescue Key Technology and Equipment, Ministry of Emergency Management, Wenzhou 325000, China

**Keywords:** blast injuries, acute lung injury, hemorrhage, inflammation, blood coagulation, therapeutics

## Abstract

Primary blast lung injury (PBLI), caused by exposure to high-intensity pressure waves from explosions in war, terrorist attacks, industrial production, and life explosions, is associated with pulmonary parenchymal tissue injury and severe ventilation insufficiency. PBLI patients, characterized by diffused intra-alveolar destruction, including hemorrhage and inflammation, might deteriorate into acute respiratory distress syndrome (ARDS) with high mortality. However, due to the absence of guidelines about PBLI, emergency doctors and rescue teams treating PBLI patients rely on experience. The goal of this review is to summarize the mechanisms of PBLI and their cross-linkages, exploring potential diagnostic and therapeutic targets of PBLI. We summarize the pathophysiological performance and pharmacotherapy principles of PBLI. In particular, we emphasize the crosstalk between hemorrhage and inflammation, as well as coagulation, and we propose early control of hemorrhage as the main treatment of PBLI. We also summarize several available therapy methods, including some novel internal hemostatic nanoparticles to prevent the vicious circle of inflammation and coagulation disorders. We hope that this review can provide information about the mechanisms, diagnosis, and treatment of PBLI for all interested investigators.

## 1. Introduction

Primary blast injury results only from the direct impact of a shockwave on the body without injuries induced by debris, wall collapse, inhalation of toxic substances, and other causes. Shockwaves can damage all organs of the body, especially the air-containing organs such as the lungs, ears, nose, stomach, and intestines [1]. Primary blast lung injury (PBLI) is defined as “radiological and clinical evidence of acute lung injury (ALI) occurring within 12 h of exposure and not due to secondary or tertiary injury” [2]. Blast lung injuries are always characterized by the absence of external signs despite severe and often lethal internal injuries; therefore, they are frequently underestimated. In severe cases, PBLI can develop into ARDS and multiple organ dysfunction syndrome, which is potentially life-threatening [3].

Although studies of PBLI in clinical observation and animal experimental models have provided a certain understanding of its main pathological manifestations, there is still a lack of treatment strategies. At present, the treatment of PBLI is based on mechanical ventilation and supportive therapy in the intensive care unit. In this review, we summarize the pathophysiological performance and pharmacotherapy principles of PBLI. In particular, we notice that the crosstalk between hemorrhage and inflammation, as well as coagulation, may represent the core mechanism, and we propose that early control of hemorrhage is the main treatment of PBLI. The cross-linkages in the mechanisms of PBLI are expected to be potential diagnostic and therapeutic targets for PBLI.

## 2. Pathophysiological Performance of PBLI

The clinical manifestations of PBLI are similar to those of pneumonia and hemorrhagic pneumonia. The specific phases and pathological manifestations are shown in Table 1. 

### 2.1. Pulmonary Hemorrhage

Shockwaves injure the body via two types of energy, namely, stress waves and shear waves [4]. Stress waves are longitudinal waves that move at supersonic speeds and create a “spalling” effect at the air–tissue interfaces, resulting in severe microvascular damage and tissue disruption [4]. The speed of transmission of stress waves through the lung does not allow gas to escape along the airways, resulting in a large amount of gas gathering into the alveoli [2]. The rapid compression and expansion of these gases in the alveoli, i.e., the implosion effect, leads to alveolar rupture manifested as pneumatoceles, lung laceration, pneumothorax, and venous air embolism [2]. Shear waves are transverse waves that cause asynchronous movement of tissue and possible disruption of attachments [4]. These principal effects cause hemorrhage in the alveoli and bronchioles.

Patients with alveolar hemorrhage may suffer from dyspnea, cough, and even hemoptysis [2,5]. In the clinic, the diagnosis of blast lung injury (BLI) is predominantly based on patients with a history of explosion exposure, with both clinically significant hypoxia and radiological evidence of blast injury to the lung seen within 12 h of exposure and not caused by secondary or tertiary injuries [2,6]. Radiologic evidence of blast lung injury mainly includes the presence of diffuse alveolar hemorrhage, which is typically bilateral and perihilar [7].

Pulmonary hemorrhage has also been confirmed in PBLI animal experiments. Li et al. [8] observed that all rats suffering from burn–blast combined injury had different degrees of lung hemorrhage. Rats with mild injury showed interstitial hemorrhage, while rats with severe injury showed alveolar hemorrhage. Moreover, the rats in the injured group showed alveolar structural disorder, red blood cell and inflammatory cell infiltration into the alveoli, alveolar septal edema, and alveolar septal thickening. In our previous studies, we also noticed mild hemorrhage and edema in PBLI mouse lungs [9].

### 2.2. Inflammation, Oxidative Stress, and Apoptosis

In addition to pulmonary hemorrhage, early inflammatory responses accompanied by increased oxidative stress and apoptosis in lung tissue occurred in mice experiencing blast injury [10].

The lung injury induced by a blast was then followed by hemorrhage and parenchymal injury, before finally reaching the inflammatory phase. Inflammatory responses are thought to play an important role in the development of blast exposure-induced lung injury [11]. Increased lung leukocyte infiltration and proinflammatory cytokine levels in the lungs are frequently associated with PBLI. Damaged lung epithelial cells release a large number of damage-associated molecular proteins, which cause the release of proinflammatory cytokines, including interleukin-1 (IL-1), IL-6, tumor necrosis factor-α (TNF-α), and C–C motif chemokine ligand 2 (CCL2) [12]. In addition, Tong et al. [10] and Wang et al. [13] observed that the expression of NF-κB, TNF-α, and other inflammatory factors in lung tissue increased significantly in BLI mouse and rat models, respectively. Inflammatory factors including TNF-α, interleukin-6 (IL-6), and IL-1β accelerate lung tissue fibrosis and respiratory dysfunction. When the release of these proinflammatory cytokines is out of control, a cytokine cascade reaction occurs, leading to tissue damage and then multiple organ failure. If inflammation is not suppressed, PBLI patients can develop ARDS with a mortality rate of 46–57% [12]. In PBLI patients, chest computed tomography (CT) scanning showed bilateral lung opacities, similarly to pneumonia [14].

In addition to inflammation, blast exposure also induced oxidative stress [6,7] and apoptosis [8] in the lungs of experimental animals. Gorbunov et al. [15] reported that blast exposure could cause oxidative stress in the lung, characterized by a decrease in total antioxidant reserves, a depletion of the antioxidant ascorbate, including vitamin E and glutathione, an increase in the products of lipid peroxidation, and an increased methemoglobin content in the lung after exposure. The mechanism of oxidative stress induced by blast exposure is still unclear. Alveolar capillary rupture leads to the emergence of free hemoglobin in lung tissue, which can induce the production of large amounts of free radicals to mediate oxidative stress injury, thereby aggravating lung injury [13,16]. This may be one of the mechanisms of oxidative stress that aggravate PBLI and even lead to its development into ARDS.

Qi et al. [17] demonstrated in rabbit BLI models that the apoptosis index, caspase-3 expression, and Bax/Bcl-2 levels were significantly increased after blast exposure. Furthermore, Liener et al. [18] found that, compared with the control group, the number of cells, neutrophil infiltration, and caspase-8 expression in the lung were significantly increased in rats suffering from blast waves. These studies demonstrated that apoptosis plays an important role in the occurrence and development of PBLI by participating in lung injury and promoting the progression of ALI/ARDS.

### 2.3. Coagulation Disorders

Clinically, coagulation disorders, as a key pathophysiological change, are responses to severe trauma injuries, and blast injury is no exception.

In a retrospective review of combat casualties, Simmons et al. [19] reported that victims of explosions or blasts, whatever the degree of injury, had deeper microcirculation impairment and more severe coagulopathy of trauma (COT) than victims of gunshot wounds. The addition of hemorrhage leading to deeper shock and more severe tissue damage in complex blast injuries could lead to a COT, as described by Brohi et al. [20] However, in blast-injured pigs, Prat et al. [21] observed that primary blast injury did not cause an acute COT in the first hour after the explosion but seemed to lead to an early hypercoagulable state. Chai et al. [22] observed a time-dependent change of coagulation function in rats with burn–blast combined injury from the parameters of blood coagulation. They found that rats suffered from hypercoagulation within 24 h and 48 to 72 h after injury. Therefore, the disorders of coagulation function in PBLI should be further researched to determine if it was secondary to other pathological changes or if it was directly caused by the explosion.

## 3. Crosstalk among Hemorrhage, Inflammation, and Coagulation

**Figure 1:**
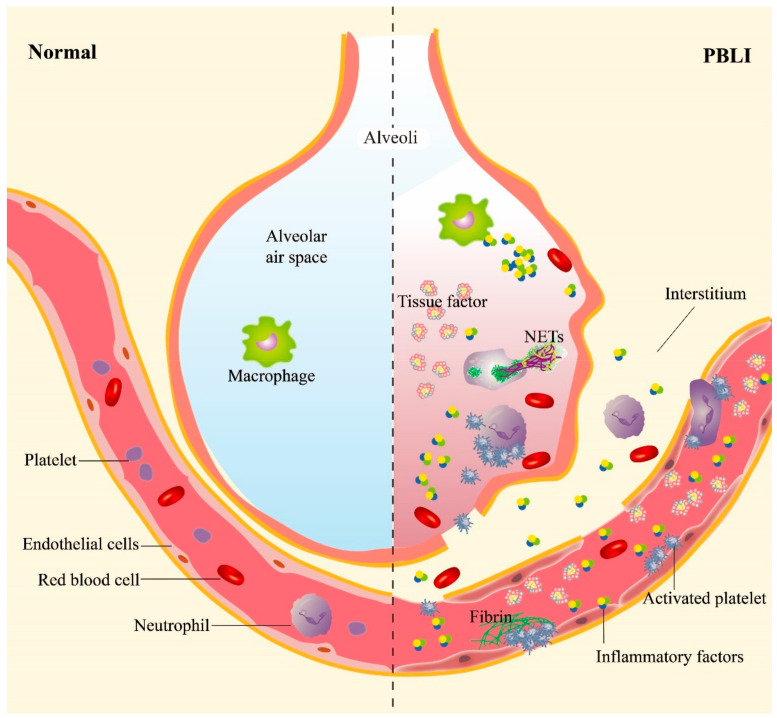
The main pathological manifestations in PBLI. The main pathological manifestations of PBLI are pulmonary hemorrhage, inflammation, and coagulation disorders. The shockwave caused rupture of the pulmonary capillaries, destruction of the alveoli, and entry of red blood cells into the alveolar space and interstitium. Inflammation is manifested by leukocyte infiltration and increased levels of proinflammatory cytokines in the lung. Coagulation disorders are characterized by the aggregation of activated platelets to form platelet thrombi and the gradual formation of fibrin thrombi. NETs: neutrophil extracellular traps.

Inflammation and hemorrhage are the two main manifestations of PBLI, subsequently inducing a series of pathological and physiological changes, such as pulmonary edema, alveolar hemorrhage, and emphysema. The processes of inflammation and hemorrhage are not independent but are related to each other (Figure 1). Shockwaves destroy the pulmonary vascular structure directly and induce the activation of platelets, which are essential for maintaining hemostasis following mechanical injury to the vasculature. Meanwhile, inflammatory responses are triggered furtherly.

### 3.1. Hemorrhage Promotes Inflammation in PBLI

The existing literature shows that shockwaves could induce platelet activation [23], expressing a variety of cell surface proteins involved in inflammation. It has been reported that P-selectin, an adhesion molecule present on activated platelets, promotes neutrophil–platelet, platelet–platelet, and monocyte–platelet interactions by binding to P-selectin glycoprotein ligand-1 (PSGL-1) on other cells [24,25].

Some studies have shown that soluble CD40L (sCD40L), as a platelet-derived microparticle, shed from the surface of activated platelets, is capable of activating leukocytes and endothelial cells [26,27]. Platelet-expressed CD40L interacts with CD40 expressed on endothelial cells, which causes numerous downstream effects, upregulating a number of proinflammatory mediators such as intracellular adhesion molecule 1 (ICAM-1), vascular cell adhesion molecule 1 (VCAM-1), and E-selectin [26].

Studies have confirmed that platelets express low levels of TLRs as pattern recognition receptors in the resting state; once activated, the expression of TLRs is upregulated, which triggers the downstream phosphatidylinositol 3-kinase (PI3K) signaling pathway to activate nuclear factor κB (NF-κB) and promote the release of inflammatory factors (TNF-α, IL-1, and IL-6), chemokines, and adhesion molecules (ICAM-1, VCAM-1, and ELAMs) [28,29]. Platelets express various TLRs, among which TLR4 plays a major role in inflammation. TLR4 has been shown to enhance platelet–neutrophil aggregations [30], and neutrophil extracellular trap (NET) formation in sepsis [30]. Wu et al. [31] observed that inhibition of the TLR4 signaling pathway could alleviate the pulmonary inflammatory response in ALI often caused by blunt chest trauma with hemorrhagic shock (THS). Thus, during the process of PBLI, we posit that TLR4 and its ligands play important roles in the post-traumatic immune response and the development of inflammation in PBLI.

**Figure 2:**
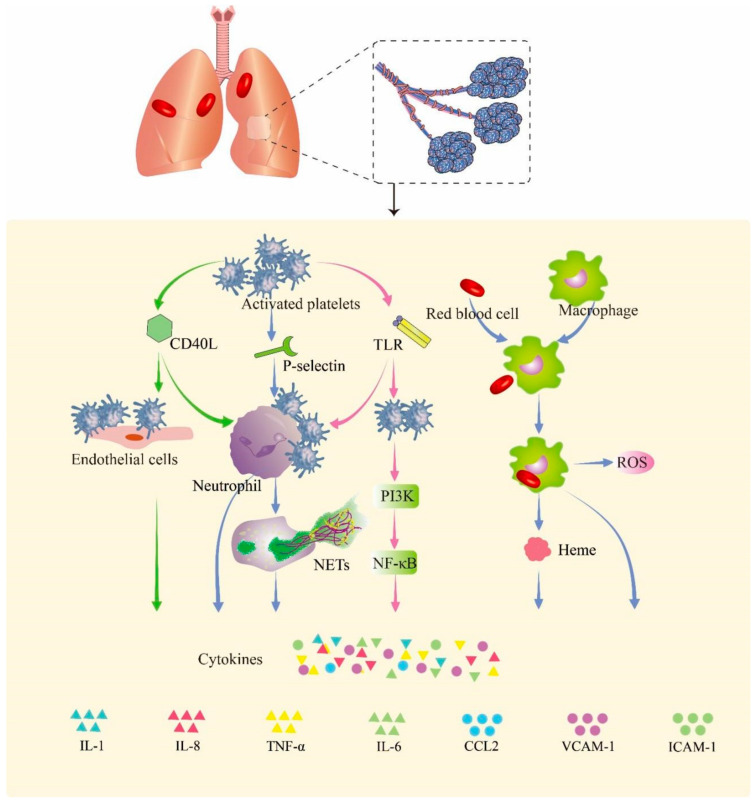
Hemorrhage promotes inflammation in PBLI. Activated platelets express a variety of cell surface proteins such as CD40Ls, P-selectins, and TLRs to trigger inflammation responses. The p-selectin expressed by activated platelets mediates the binding of platelets to neutrophils. CD40L mediates the binding of platelets to endothelial cells and neutrophils. The expression of TLRs in platelets promotes binding with neutrophils. Upon binding, neutrophils, endothelial cells, and activated platelets initiate an inflammatory response that promotes the release of cytokines. The red blood cells that leak into the alveoli are swallowed by macrophages and release heme and globulin. Heme induces the release of ROS and activation of the NF-κB signaling pathway in macrophages, thereby triggering inflammation. CD40L: cluster of differentiation 40 ligand; NETs: neutrophil extracellular traps; TLR: Toll-like receptor; PI3K: phosphoinositide 3-kinase; NF-κB: nuclear factor kappa B; ROS: reactive oxygen species; IL-1: interleukin-1; IL-8: interleukin-8; TNF-α: tumor necrosis factor-α; IL-6: interleukin-6; CCL2: C–C motif chemokine ligand 2; VCAM-1: vascular cell adhesion molecule 1; ICAM-1: intercellular adhesion molecule 1.

After blast injury, large numbers of hemoglobin-containing red blood cells leak into the alveoli, swallowed by macrophages in the alveoli, and heme is subsequently released from hemoglobin [32]. Free heme, as a metabolite after the rupture of red blood cells, can induce the proinflammatory response of macrophages. It can induce the production of reactive oxygen species (ROS) and NF-κB signaling molecules in macrophages, thereby promoting the release of inflammatory factors [33]. In Figure 2, we summarize the probable pathway of hemorrhage promoting inflammation in PBLI.

### 3.2. Inflammation Aggravates Coagulation Disorders

**Figure 3:**
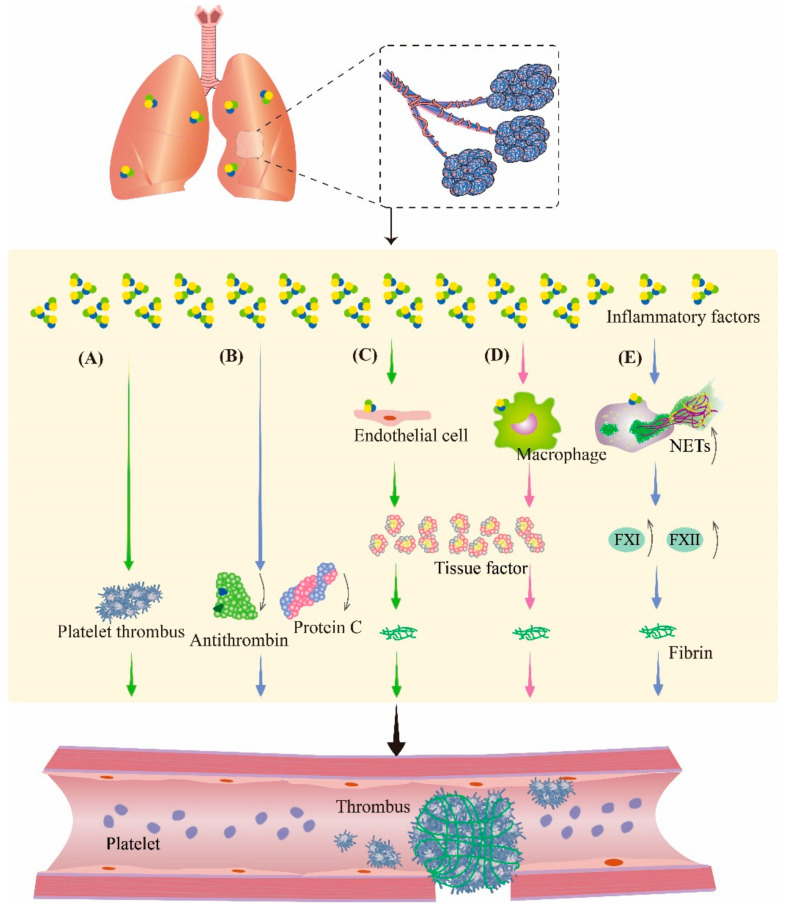
Inflammation aggravates coagulation disorders in PBLI. During inflammation, inflammatory factors can promote thrombosis by regulating the coagulation system and fibrinolytic system. (**A**) Inflammatory factors promote platelet activation and aggregation to form platelet thrombosis. (**B**) Inflammatory factors cause the downregulation of antithrombin and protein C to inhibit fibrinolysis. (**C**,**D**) Inflammatory factors stimulate endothelial cells and monocytes to produce tissue factor, an activator of coagulation, resulting in fibrinous thrombi. (**E**) Neutrophils are activated by inflammatory factors to produce NETs, which promote coagulation by promoting the production of several coagulation factors. NETs: neutrophil extracellular traps; FXI: coagulation factor XI; FXII: coagulation factor XII.

The inflammatory response is a multifactorial defensive process of an organism to injury, such as infectious or noxious stimuli. During inflammation, cytokines modulate the coagulation system; therefore, in the study of inflammation, the involvement of the coagulation pathway must be taken into account. Therefore, it is speculated that inflammation is also crosslinked with coagulation during PBLI (Figure 3). These changes make the management of pulmonary hemorrhage after PBLI more complicated, which deserves further study.

Proinflammatory cytokines including interleukins and tumor necrosis factor (TNF) directly promote local coagulation, in addition to being responsible for regulating inflammatory responses [34]. A previous study [35] showed that the exposure of whole blood from healthy volunteers to IL-1β, IL-6, and interleukin-8 (IL-8) resulted in hyperactivation of platelets and increased clotting. In addition, TNF promotes platelet aggregation and activation. Several studies also confirmed that proinflammatory cytokines such as TNF-α, IL-1, and IL-6 play important roles in the initiation of coagulation [36,37,38,39]. In addition to promoting coagulation through the above pathways, proinflammatory cytokines also cause obstacles to the anticoagulant mechanism by inhibiting the activity of antithrombin (AT) and protein C [38].

TF is expressed on platelets, endotheliocytes, neutrophils, and eosinophils at organ and body surfaces. As the initiator of the extrinsic coagulation pathway, TF plays a central role in inflammation-induced coagulation initiation [40]. Normally, TF is hardly present in the circulating blood. However, in an inflammatory state, monocytes and endothelial cells can increase the expression of TF through proinflammatory cytokines such as TNF-α, IL-1β, and IL-6 [36,41]. The TF expressed by monocytes and neutrophils promotes coagulation in turn.

After activation by cytokines or cytotoxins, neutrophils produce an extracellular fibrous network of DNA, histones, and granulins such as elastase to capture bacteria. This network of extracellular fibers is known as neutrophil extracellular traps (NETs). NETs have been reported to be associated with platelet aggregation and coagulation [42]. Fuchs et al. [42] discovered that NETs provide a scaffold and stimulus for platelet binding and aggregation. Histones in NETs or liberated after digestion of NET can also provide a stimulus for platelet aggregation. Cell-free DNA (cfDNA), as a component of NETs, promotes thrombosis by activating some proteases in the coagulation pathway, such as coagulation factors XII and XI, and suppresses fibrinolysis [43]. In our previous study [9], we found elevated expression of NETs in the lungs of mice with PBLI, which indicated an increased thrombotic risk in PBLI. After blast exposure, due to the overexpression of proinflammatory factors, TF, and NETs, local coagulation in lung tissue may result, which is contradictory to the pulmonary hemorrhage reaction, further complicating the treatment of PBLI.

## 4. Pharmacotherapy Principles for PBLI

At present, the clinical treatment of PBLI is mainly based on mechanical ventilation, intensive treatment, and supportive treatment. Pharmacotherapy methods that target different pathological manifestations of PBLI to stop bleeding, inhibit inflammation, and stabilize coagulation may be used to treat PBLI (Table 2).

### 4.1. Hemostasis

Following an explosion accident, uncontrollable internal hemorrhage is the main cause of high mortality of the injured. If the hemorrhage is not inhibited, it can cause serious respiratory dysfunction and even systemic hemorrhagic shock [57]. Hemorrhagic deaths are potentially preventable if hemorrhage can be controlled in a timely manner [58]. Therefore, early control of hemorrhage is the key for PBLI.

#### 4.1.1. Recombinant Activated Factor VII (rFVIIa)

Recombinant activated factor VII (rFVIIa) was developed in the 1980s for patients with hemophilia because of its role in the activation of coagulation. As a coagulation factor, FVIIa can interact with TF expressed in the vascular endothelium to promote the production of thrombin, initiating the body’s coagulation process and thus achieving the purpose of hemostasis [44]. Many case reports [59,60,61,62] have suggested the efficacy of rFVIIa in the treatment of diffuse alveolar hemorrhage (DAH) from a range of medical causes. Estella et al. [5] reported two patients admitted to the ICU with DHA in whom bleeding stopped immediately after bronchoscopic administration of rFVIIa, and no thrombotic complications were observed thereafter. A retrospective database analysis [6] showed that the most common indications for rFVIIa use were adult and pediatric cardiovascular surgery (29%; CI: 21% to 33%), body and brain trauma (29%; CI: 19% to 38%), and intracranial hemorrhage (11%; CI: 7.7% to 14%). Over previous decades, the use of rFVIIa in trauma patients became widespread.

In view of the intrapulmonary hemorrhage caused by PBLI, some researchers proposed to promote hemostasis using rFVIIa [57]. There are two administration routes of rFVIIa: intrapulmonary and intravenous. Similar to most biologics, rFVIIa requires a high systemic concentration to affect specific receptors in the alveolar compartment, which requires higher and repeated doses of intravenous rFVIIa. Therefore, intravenous administration could cause adverse effects associated with a higher risk of thromboembolic [57]. Intrapulmonary administration of rFVIIa is recommended as the treatment of choice for DAH and blast lung injury (BLI) because the treatment has been shown to be successful and uncomplicated [57]. Therapeutic effects of aerosol inhalation of nebulized rFVIIa have also been demonstrated in BLI and other studies characterized by DAH [44,63,64,65]. However, Smith [66] showed different effects of rFVIIa on alveolar hemostasis in a rabbit model of BLI. This study did not provide any evidence that a single dose of nebulized rFVIIa (200 µg/kg total dose deposited in the lungs) 30 min after blast exposure attenuates the hemorrhagic element of BLI. There were no significant differences in hemostasis between the rFVIIa and placebo treatment groups over the duration of the study. We suspect that the reason for the divergence in the effects of rFVIIa in PBLI may have been the single dose used in the experiment.

#### 4.1.2. Tranexamic Acid (TXA)

Hyperfibrinolysis is associated with uncontrolled bleeding due to clot dissolution, and it is associated with high bleeding-related mortality [67]. Chai et al. [22] found that rats with burn–blast combined injury had severe lung injuries with a secondary excessively fibrinolytic function. Therefore, anti-fibrinolysis is crucial for the treatment of PBLI.

Tranexamic acid (TXA) is believed to inhibit plasminogen activation by binding to kringle domains, thus preventing rearrangement to the disulfide-linked, two-chain, active plasmin form. As an anti-fibrinolytic agent, TXA can block the lysine binding site of plasminogen, thereby inhibiting plasminogen activation and fibrinogen–plasminogen binding. Fibrinolysis is impaired, clot decomposition is inhibited, and hemorrhage is reduced [68,69]. Previous studies have shown that TXA can significantly improve bleeding in trauma patients. Roberts [45] suggested that initiation of TXA treatment within 3 h of injury reduces the risk of hemorrhage death by approximately one-third, regardless of baseline risk, with no increase in vascular occlusive events in trauma. In a multicenter, randomized, and placebo-controlled trial (CRASH-2 trial), a total of 20,211 adult trauma patients, with or at risk of significant bleeding, who were within 8 h of their injury, were randomly allocated to receive TXA or a matching placebo. The primary outcome was death within 4 weeks. TXA significantly reduced death due to bleeding and all-cause mortality [70].

The collateral beneficial effects of TXA beyond direct anti-fibrinolysis may be explained by its modulatory effect on inflammation [71]. For example, Carter et al. [72] found that TXA reduced lung inflammation as represented by decreased macrophage infiltration, and TXA may be a useful adjunct in burn resuscitation. In a controlled hemorrhagic shock rat model, Walker et al. [73] reported that TXA could significantly inhibit the expression of inflammatory cytokines and improve pulmonary edema. Therefore, TXA can not only reduce post-traumatic bleeding, but also inhibit inflammation. As PBLI patients also exhibit bleeding and inflammation, TXA may also be a potential treatment for PBLI.

However, in some research, TXA was found to have prothrombotic effects. In a study of traumatic brain injury (TBI), where most casualties were injured in a blast (80.3%), and 10.3% of patients had intracranial hemorrhage (ICH), researchers evaluated the association between TXA use and progression of ICH and venous thromboembolism (VTE). They found that patients administered TXA had lower initial reported Glasgow Coma Scores (GCS), but a higher rate of VTE. The results of this study suggest that TXA, used as an adjunct for military patients with severe TBI, may be involved in the risk of thrombotic events. Therefore, TXA can be used to reduce traumatic hemorrhage and reduce the mortality of traumatic hemorrhage, and treatment with TXA can be implemented in clinical practice as part of a strategy for severe wartime injuries and post-hemorrhage resuscitation.

PBLI, characterized by alveolar hemorrhage, has similar pathological manifestations to TBI. Hence, TXA therapy for PBLI has rationale, but whether it will lead to an increase in thrombotic events is still worthy of future study.

#### 4.1.3. Novel Internal Hemostatic Nanoparticles

Several research teams have developed a variety of new nanoparticles with hemostatic functions. By attaching a substance or peptides, the nanoparticle can be endowed with the function of targeting to the injury site, which can greatly improve its therapeutic effect. Here, we summarize three representative hemostatic nanoparticles.

Fibrinogen γ-chain-coated adenosine 5′-diphosphate-encapsulated liposomes (H12-(ADP)-liposomes) bind to GPIIb/IIIa on the surface of activated platelets through H12, such that the liposome is targeted to the injured site to inhibit internal bleeding [46]. It can promote platelet aggregation and tissue protection through ADP, a physiological platelet agonist [46]. In a rat model of thrombocytopenia induced by ibuprofen, it was found that the tail bleeding time in mice treated with H12-(ADP)-liposomes was significantly reduced compared with the control group [74]. Hagisawa et al. [46] also showed that this liposome increased the survival rate from 8% to 58% in mice suffering from lethal BLI. H12-(ADP)-liposomes may be an effective therapy strategy against BLI by promoting tissue-protective adenosine signaling.

The main components of thrombin@Fe_3_O_4_ nanoparticles are Fe_3_O_4_ and thrombin [75], which target the damaged site through a magnetic field and promote the coagulation cascade through thrombin [76]. The researchers used human plasma for coagulation tests and found that the coagulation time of blood treated with nanoparticles was reduced twofold compared to thrombin, meaning that the nanoparticle was more effective in hemostasis.

Hubbard et al. [77] synthesized hemostatic dexamethasone nanoparticles (hDNPs) to alleviate internal hemorrhaging following a traumatic event. They consisted primarily of a block copolymer, poly (lactic-co-glycolic acid)–poly(l-lysine)–poly (ethylene glycol) conjugated to a peptide, glycine–arginine–glycine–aspartic acid–serine (GRGDS) and dexamethasone. This nanoparticle could accumulate at the bleeding site through GRGDS targeting activated platelets, while dexamethasone exerted an anti-inflammatory effect [77]. In a rat PBLI and TBI model, Hubbard’s team [47] found that intravenous administration of hDNPs would reduce hemorrhage and inflammation and increase the survival rate from ~60% to ~80% after blast trauma.

### 4.2. Anti-Inflammatory Therapy

Inflammation is involved in the second stage of PBLI, at 4–12 h [57]. Uncontrolled inflammation causes continuous self-amplification, which further leads to leukocyte aggregation, resulting in epithelial cell damage, endothelial cell damage, and eventually typical ARDS. Therefore, suppressing inflammation is crucial for PBLI therapy. At present, some anti-inflammatory treatments for PBLI have been applied to clinical treatment. This section reviews some potential anti-inflammatory treatments for PBLI, in order to provide new ideas.

#### 4.2.1. Inhibitors of Neutrophil Elastase (NE)

Neutrophil elastase (NE), stored in the azurophil granules of neutrophils, is involved in a variety of physiological processes such as the formation of NETs and the degradation of extracellular matrix and proteins, and it plays important roles in the pathogenesis of ARDS [78]. NE facilitates leukocyte transmigration to the site of infection and activates inflammation directly by increasing cytokine expression and release [79]. NE induces monocyte–macrophage cells to produce IL-8, a cytokine that promotes neutrophil adhesion and exudation, which results in the accumulation of neutrophils in lung tissue [80]. Xiao et al. [48] observed that the expression of NE in serum, bronchoalveolar lavage (BAL), and lung tissue of severely burned rats was significantly higher than that of the control group. The newly recruited neutrophils release more NE, which results in a vicious cycle, thereby aggravating inflammation and pulmonary edema. Indeed, as discussed below, NE inhibitors have shown good therapeutic effects on ARDS and even PBLI in animal and clinical trials.

Sivelestat is a synthetic specific low-molecular-weight NE inhibitor that can competitively inhibit neutrophil elastase activity but does not affect other serine proteases released by polymorphonuclear (PMNs) [81]. At present, sivelestat has been applied to the clinical treatment of ARDS. Lee et al. [82] also confirmed that sivelestat could alleviate pulmonary edema and histopathological changes in LPS-induced ALI mice, as well as inhibit the aggregation of neutrophils and release of TNF-α, IL-6, and MPO in bronchoalveolar lavage fluid (BALF). Sivelestat has also been shown to be effective against inflammation in severe burns. Xiao et al. [48] found that sivelestat could reduce the expression of NE and IL-8 in serum and bronchoalveolar lavage fluid, as well as reduce the expression of matrix metalloproteinase (MMP) 2 and 9 in rat models of severe burns.

Ulinastatin is a glycoprotein that can inhibit the activity of various proteolytic enzymes. It has been used to treat acute pancreatitis, acute circulatory failure, and severe sepsis in the clinic [83]. Furthermore, studies have found that ulinastatin also has a good therapeutic effect in ALI. Cao et al. [84] found that ulinastatin ameliorates LPS-induced inflammation by attenuating TLR4/NF-κB pathway activation, thereby increasing the survival rate of ALI from 20% to 50%. In rats with severe burn–blast combined injury, ulinastatin reduced the infiltration of inflammatory cells, thus alleviating lung injury [49]. In a rabbit PBLI model, ulinastatin can significantly reduce the acidosis of ALI rabbits caused by blast injury, reduce PaCO_2_, pulmonary edema, and neutrophil infiltration, and increase PaO_2_ and the oxygenation index [50]. Ulinastatin combined with dexamethasone, the most commonly used long-acting glucocorticoid in clinical practice that has a strong anti-inflammatory effect, has a certain therapeutic effect on ALI caused by blast injury in rabbits, with the effect significantly better than that of ulinastatin alone [51]. In addition, some studies have shown that ulinastatin also has certain anticoagulant properties [85,86].

#### 4.2.2. Perfluorocarbon

Perfluorocarbon (PFC), as a colorless and odorless liquid composed of fluorine and carbon, has high solubility of oxygen and carbon dioxide and has been proven to play an effective role in ALI/ARDS [52]. Partial liquid ventilation (PLV) using perfluorocarbon (PFC) has been proven to improve gas exchange and respiratory dynamics of the lungs during ARDS [87]. Moreover, studies have shown that liquid ventilation with PFCs protects the lung from acute inflammation by reducing the neutrophil infiltration in different lung injury animal models and clinical studies. PFC-based treatment modalities have been shown to reduce the levels of cytokines, chemokines, and other mediators of pulmonary inflammation [88].

Wei et al. [87] proved that PFC liquid ventilation-induced mild hypothermia improved the lung function and alleviated the inflammatory response in canines with ARDS induced by oleic acid infusion. In vivo and in vitro models of PBLI and PFC also showed a therapeutic effect. Zhang et al. [52] demonstrated that in the PBLI cell model, PFC treatment could inhibit proinflammatory cytokine release and oxidative stress, which may be achieved by regulating the expression of IL-6 and TNF-α through NF-κB and MAPK pathways. In PBLI animal models, Zhang et al. [53] proved for the first time that vaporization PFC could reduce the inflammation response in a canine BLI model, and that PFC had a particularly potent anti-inflammatory effect by suppressing NF-κB and MAPK activation and the Nrf2 pathway. Therefore, vaporization PFC may be used to solve the inflammation during ALI/ARDS, including PBLI.

#### 4.2.3. N-Acetylcysteine and N-Acetylcysteine Amide

N-Acetylcysteine (NAC) and N-acetylcysteine amide (NACA) are both antioxidants [89], and also have anti-inflammatory effects. In endotoxic shock animals, NAC decreased neutrophil-aggregating activity, reduced pulmonary hypertension, and attenuated vascular permeability. It has been reported that NAC and NACA play essential roles in ALI/ARDS. Kao et al. [90] found that NAC obviously reduced the mortality from 43% to 23% in ARDS rats induced by LPS. Jansson et al. [91] found that NAC pretreatment could inhibit TNF-α production to some extent in an LPS-induced ALI rat model. Assimakopoulos et al. [92] showed that oral NAC administration (1200 mg/day) in patients with COVID-19 pneumonia reduced the risk of mechanical ventilation and mortality, as NAC improved the PO2/FiO2 ratio over time, as well as decreased the white blood cell, C-reactive protein, D-dimer, and lactate dehydrogenase levels. In a PBLI rat model, Chavko et al. [54] reported that NACA significantly decreased myeloperoxidase activity in lung tissue, which is an indicator of neutrophil infiltration, and the administration of both NAC and NACA resulted in a significant reduction in NF-κB activation and attenuated lung inflammation caused by increased production of ROS. Thus, NAC could reduce inflammation in PBLI by inhibiting neutrophil aggregation, NF-κB, and TNF-α production.

### 4.3. Anticoagulation in PBLI

#### 4.3.1. Heparin

Heparin is a member of a family of glycosaminoglycan molecules that include heparan sulfate, chondroitin sulfate, keratan sulfate, and hyaluronic acid. These molecules are expressed throughout the body, with diverse biological roles. Heparin’s anticoagulant properties have been used in clinical practice to limit systemic fibrin deposition since 1935. Heparin inhibits coagulation activation through a range of mechanisms, including catalyzing the action of antithrombin, promoting tissue factor pathway inhibitor (TFPI) expression, reducing tissue factor expression, and increasing endothelial expression of heparan sulfate, as well as through release of tissue plasminogen activator by the endothelium.

Nebulized heparin has many advantages over systemic administration, which targets pulmonary fibrin deposition and inflammation, reducing the risk of systemic bleeding. It allows higher dosages and has better local efficacy. Previous studies have shown that nebulized heparin rarely enters the systemic circulation after inhalation, which means that nebulized heparin treatment has a low risk of systemic bleeding [93].

The existing literature shows that the anticoagulant effect of nebulized heparin limits fibrin deposition and microvascular thrombosis, as well as inhibits coagulation in COVID-19 [93,94]. For the treatment of pulmonary coagulation in BLI, an actual effective therapy strategy is still lacking, which is worth exploring in the future. Chai et al. [95] confirmed that diffuse intravascular coagulation was prevented by using low-molecular-weight heparin (5000 U 2–4 times/day) in severe burn–blast combined injury patients with a positive plasma protamine paracoagulation test and abnormal coagulation state.

However, whether heparin can increase bleeding in patients with traumatic hemorrhage (TH) is still controversial, which restricts the application of heparin in TH patients. Fu et al. [96] showed that patients with major orthopedic surgery or trauma who received subcutaneous injection of low-molecular-weight heparin had a risk of bleeding, which was characterized by a 1.5% probability of in-hospital major bleeding and a 26% probability of in-hospital minor bleeding. In rats suffering from blast injury and hemorrhagic shock, Yang et al. [55] studied the roles of extracellular high-mobility group box 1 protein (HMGB1, also known as amphoterin) and its inhibitor, CX-01 (2-O,3-O-desulfated heparin), one of the minimally anticoagulant desulfated heparins, which does not cause heparin-induced thrombocytopenia but retains the anti-inflammatory properties. They found that extracellular HMGB1, released from damaged/necrotic tissue and the activated innate immune system, has a strong proinflammatory effect. Early CX-01 treatment could improve survival and significantly attenuate TH-induced lung injury [55]. Taken together, their clinical and preclinical findings show that HMGB1 plays a critical role in the pathogenesis of trauma and hemorrhage, and that CX-01 may be a promising pharmacological solution for the treatment of severely injured patients in prehospital settings.

#### 4.3.2. rTFPI

Tissue factor pathway inhibitor (TFPI) is an alternatively spliced anticoagulant protein present in plasma, platelets, and the extracellular matrix, as well as on the surface of endothelial cells, monocytes, and macrophages. It is a multifunctional Kunitz-type serine protease inhibitor that acts at several steps of the blood coagulation cascade [97]. TFPI primarily inhibits the initiation of coagulation, dampening procoagulant stimuli before thrombin is generated [98]. The anticoagulant effect of TFPI can be attributed to two mechanisms: inhibition of tissue factor-activated coagulation factor VII (TF-FVIIa) and activated coagulation factor X (FXa), via the tissue factor-activated coagulation factor VII/tissue factor pathway inhibitor-activated coagulation factor X (TF-FVIIa/TFPI-FXa) complex, and inhibition of prothrombinase, as reported in a recent study [99,100]. De Jonge et al. [98] studied the therapeutic effect of intravenous TFPI on healthy volunteers after intravenous endotoxin administration. The results showed that intravenous injection of TFPI increased the levels of thrombin/antithrombin complexes and prothrombin fragment in blood, but did not change the contents of tPA, PAI-1, or PAPc, or the contents of TNF-α and IL-6, which meant that endotoxin-induced thrombin production was blocked and coagulation activation was inhibited. Moreover, the effect of TFPI on thrombin generation was dose-dependent, and TFPI had no effect on fibrinolysis or cytokine release. Nebulized TFPI has also shown good effect in lung injury. For example, the authors of [101] reported that nebulized TFPI reduced pulmonary coagulopathy in animals with a pulmonary infection or lung injury. Local treatment with recombinant human TFPI did not alter systemic TFPI activity; however, it attenuated both pulmonary and systemic coagulopathy [101]. Furthermore, nebulized rh-TFPI modestly reduced the pulmonary inflammatory response and allowed increased bacterial clearance in rats with direct lung injury caused by P. aeruginosa.

Recently, Tian et al. [56] reported that, in a rat model with gas explosion injury, TFPI increased 2 h and 24 h after injury but decreased significantly after 72 h and returned to normal levels, indicating that the expression of TFPI changed after blast injury. As nebulized rh-TFPI plays an important role in anticoagulant and anti-inflammatory activities, it is a potential drug for the treatment of PBLI.

## 5. Conclusions and Perspectives

Blast injuries induced by accidental explosion are common in various fields such as military, industry, and daily life. Medical practitioners should strengthen their understanding of PBLI to successfully provide ameliorative and preventative services to patients. At present, treatment of PBLI remains supportive, with current best practices and specific diagnoses also lacking. It should be noted that real-time monitoring is particularly important for diagnosis and treatment at different stages of PBLI. Here, we reviewed almost all studies on PBLI at home and abroad, and we summarized the pathological presentations and drug therapy of PBLI, in order to provide new bases for its clinical treatment. However, there have been few studies on PBLI, and the number of studies related to the pharmacotherapies of PBLI is even lower, with most of them involving animal models. Therefore, these treatment strategies have certain limitations and need to be improved.

The pathological presentations of PBLI mainly involve pulmonary hemorrhage, inflammation, and potential clotting disorders, which crosstalk with each other. In PBLI patients, hemorrhage promotes pulmonary inflammation, which in turn aggravates coagulation disorders. These crosslinks always result in a dilemma faced by doctors during the process of medical treatment. Patients with severe PBLI are often at risk of secondary thrombogenesis due to their hypercoagulable state, but treatment with anticoagulants may promote alveolar hemorrhage. Among these, inflammation plays a key role in promoting both hemorrhage and coagulation disorders. Therefore, drugs that have both hemostatic and anti-inflammatory effects, such as TXA, or have both anticoagulant and anti-inflammatory effects, such as heparin, are promising for the treatment of PBLI. In addition, for anticoagulant or hemostatic treatment, atomization is a promising technique for local pulmonary administration. Biomaterials can integrate a variety of therapeutic drugs to achieve anti-inflammatory, hemostatic, or anticoagulant effects without increasing drug dosage and inducing fluid resuscitation overload. However, improvements in their pulmonary targeting and localization are future research directions.

With the increase in industrial accidents and detonation of terrorist devices, an understanding of the mechanisms of PBLI and their management should be of assistance in the aftermath of such events. Hence, we hope that doctors can gain information from this article and devote themselves to identifying specific treatment modalities for PBLI patients.

## Figures and Tables

**Table 1:** The phases, timing, and corresponding pathological manifestations of PBLI [1].

Phases and Timing	Main Pathological Manifestations of PBLI
Phase 1 [2] 0–3 h	Pulmonary hemorrhage
	The lung and blood vessels are compressed by the shockwave, forcing blood or air out through the alveolar septum or capillary walls, resulting in pulmonary hemorrhage
Phase 2 [3] 4–24 h	Inflammation
	Leukocyte infiltration and proinflammatory cytokine levels in the lungs are increased The accumulation of MPO in the injured lung gradually increases in a time-dependent manner from 3 h through 24 h
Phase 3 [4] After 24 h	Hypercoagulation
	Platelet thrombi and fibrin thrombi are formed

**Table 2:** Pharmacotherapies for primary blast lung injury.

Drug/Efficacy	Drug/Strategy	Protective Mechanism	Model	Author	Author Country	Journal Year	References
Hemostasis	Recombinant activated factor VII (rFVIIa)	Coagulation factor, promotes the production of thrombin	BLI patients	Martinowitz et al.	Israel	2004	[44]
	Tranexamic acid (TXA)	Anti-fibrinolytic agent, impairs fibrinolysis, inhibits clot decomposition	Adult trauma patients	Roberts	UK	2015	[45]
	Fibrinogen γ-chain-coated adenosine 5′-diphosphate-encapsulated liposomes(H12-(ADP)-liposomes)	Targets the injured site, inhibits internal bleeding	BLI mice	Hagisawa et al.	Japan	2016	[46]
	Thrombin@Fe_3_O_4_ nanoparticles	Targets the damaged site, promotes the coagulation cascade	-	-	-	-	-
	Hemostatic dexamethasone nanoparticles (hDNP)	Targets the bleeding site, exerts anti-inflammatory effects	BLI rats	Hubbard et al.	US	2018	[47]
Anti-inflammation	Sivelestat sodium hydrate (sivelestat)	Reduces the expression of NE and IL-8	Severe burns rats	Xiao et al.	China	2016	[48]
	Ulinastatin	Reduces the infiltration of inflammatory cells, reduces pulmonary edema and neutrophil infiltration, alleviates lung injury	Rats with severe burn–blast combined injury	Liu et al.	China	2018	[49]
			BLI rabbits	Yuan et al.	China	2016	[50]
			BLI rabbits	Dai et al.	China	2015	[51]
	Perfluorocarbon (PFC)	Inhibits proinflammatory cytokine release and oxidative stress	BLI cells	Zhang et al.	China	2017	[52]
			BLI canine	Zhang et al.	China	2020	[53]
	*N*-Acetylcysteine amide (NACA)	Decreases myeloperoxidase activity, reduces NF-κB activation, attenuates lung inflammation	PBLI rats	Chavko et al.	US	2009	[54]
Anticoagulation	Heparin	Prevents diffuse intravascular coagulation, improves survival	Trauma patients and blast injury rats	Yang et al.	US	2022	[55]
	rTFPI	Inhibits coagulation cascade	Gas explosion rats	Tian et al.	China	2020	[56]

## Data Availability

Not applicable.
